# Towards an equitable digital public health era: promoting equity through a health literacy perspective

**DOI:** 10.1093/eurpub/ckz166

**Published:** 2019-11-18

**Authors:** Natasha Azzopardi-Muscat, Kristine Sørensen

**Affiliations:** 1 Department of Health Services Management, University of Malta, Msida MSD 2080, Malta; 2 European Public Health Association, Utrecht, PO Box 1568, 3500 BN, The Netherlands; 3 Global Health Literacy Academy, Viengevej 100, 8240 Risskov, Denmark; 4 International Health Literacy Association, IHLA 7 Avenue De Lafayette 121026, Boston, MA 02112, USA

## Abstract

Digital technologies shape the way in which individuals and health systems interact to promote health and treat illness. Their propensity to exacerbate inequalities is increasingly being highlighted as a concern for public health. Personal, contextual and technological factors all interact and determine uptake and consequent use of digital technologies for health. This article reviews evidence on the impact of digital technologies on health equity. Health literacy is presented as a lens through which to approach research and policy on access, uptake and use of digital technologies. In the short term, based on our review of published literature, we conclude that it is likely that digital technologies will increase health inequities associated with increased age, lower level of educational attainment and lower socio-economic status. Geographical inequity may increase as a result of poor infrastructure but may decrease if digital technologies can be effectively widely deployed to compensate for health workforce and health system deficiencies. Programmes to enhance health and digital literacy and monitoring of access, utilization and impact across all groups in society can help to ensure that digital technologies act to reduce rather than reproduce or exacerbate existent health inequalities.

## Introduction

The ‘digital transformation of health services’ is viewed as an important and influential process, that is already exercising a substantial impact on health systems and is undoubtedly set to fundamentally alter the future of health systems.^1^^,^^2^ Just as banking, retail or travel now occur in a fully digital world without the ‘e’ prefix, this revolution will arrive to health systems. However, the impact of digitalization goes beyond the platforms and mechanisms through which patients interact with health services.^3^ The proliferation of health related smart phone applications, the quantified self-movement (a form self-knowledge through self-measurements of physiological variables) and the use of big data that draws upon health and lifestyle information all have a profound impact to shape the health of current and future generations outside the parameters of healthcare delivery.^4^^,^^5^

The World Health Organization classifies ‘digital technologies’ used for health and health services into four distinct categories^6^:

- Interventions for clients.
- Interventions for healthcare providers.
- Interventions for health system or resource management.
- Interventions for data services.

Although all of these interventions can have an impact on equity in health, the main focus of this paper pertains to direct interventions of digital technologies for clients (citizens, patients, careers).

A commonly held aspiration is that:

**‘** *Now that digital technologies have provided almost full interconnectivity among all humans, they should be used to meet key challenges to ensure that health is created and that it spreads to reach every person on earth* **’** Jimenez-Marroquin, Deber and Jadad**.**^7^

One prevailing view is that the potential to continue to improve health and empower citizens in becoming active custodians of their own health will usher in a new paradigm with previously unimaginable opportunities for health improvement. For example, opportunities may be created in some traditionally underserved groups where smartphone ownership and usage may often exceed penetrance rates in the general population.^8^^,^^9^ Others caution that there is a darker aspect that comes with the digitalization of health, namely that of increasing inequities as digitalization in health care normally affects certain goals or certain groups positively, while at the same negatively affecting others. Indeed, despite growing use of the Internet, access to health information remains unequal.^10–12^

In this paper, we seek to examine the impact that digital technologies have on equity by asking the following questions:

- How may innovative health technologies reduce or (re)produce social inequalities in health?
- How do existing studies explain the potential relationship between innovative health technology and social inequalities in health?
- What are the implications of using different measurements of social inequality on the results observed?

We propose the health literacy concept^13^ as a lens through which to approach policy in the digital health arena as a means to ensure the transformation of health systems that deliberately seek to use digital health as a tool to combat health inequalities. Finally, we set out proposals for a research agenda in this area of health equity and digitalization from a public health perspective.

### The relationship between innovative health technology and social inequalities in health

In 2016, Chauvin et al. observed that there was a paucity of research and analysis on the impact of digital technology use on population health outcomes. Furthermore, the impact of social determinants of health on the uptake, use and effects of digital technology on human health was not adequately addressed in the literature and ultimately the evaluation of the potential impact of digital technology on population health outcomes and health equity was missing.^14^

Currently many countries in Europe are already experiencing increasing relative and absolute health inequalities.^15^^,^^16^ These are often unrelated to health care and have far more to do with the political determinants of health and the shrinkage of middle-income jobs to be replaced with high and low earning jobs.^17^ In response, WHO Europe identified five essential conditions for living a healthy life and developed a corresponding policy tool to measure countries’ improvement on health equity^18^:

- Good-quality and accessible health services.
- Income security and an appropriate, fair level of social protection.
- Decent living conditions.
- Good social and human capital.
- Decent work and employment conditions.

According to the WHO ‘health equity’ or ‘equity in health’ implies that ideally everyone should have a fair opportunity to attain their full health potential and that no one should be disadvantaged from achieving this potential.^17^ However, socio-demographic characteristics—particularly age, education, income, perceived health and social isolation—also predict Internet access. Thus, in addition to widening access, the movement towards digitization of health information and services should also consider digital skills development in order not to further widen inequalities, as well as to minimize the effects of harmful digital marketing on health behaviour.^19^^,^^20^

### The mechanisms through which innovative health technologies reduce or (re)produce social inequalities in health

In a scoping review on the link between innovative technologies (not limited to digital technologies) and social inequalities in health conducted by Weiss et al. it was highlighted that in addition to the factors and mechanisms that influence unequal access to technologies, variations in use also importantly shape social inequalities in health. This is a useful starting point to understand the pathways through which various innovative health technologies reduce or (re)produce social inequalities in health. Context, baseline penetration levels, cultural attitudes and infrastructure around digital technology can all play an important role in mediating the outcome with regards to health equity and are at least as important as individual factors.^21^

### Personal and contextual factors

The difficulty in making an assessment on the relationship between diffusion and uptake of digital technologies associated with health and the effects on healthy equity at population level is partly a result of the diverse social determinants of health that may be studied leading to different results.

A review by O’Connor et al., which sought to identify key factors that determine how patients and the public engage with and enroll in a broad range of digital health interventions, found that the level of education attained, poor computer skills, literacy skills, age and ability to pay for the technology all play a role in determining uptake and use of digital health technologies.^22^ Its findings were complementary to an earlier review by Hardiker and Grant, which found that age, ethnicity, economic status and educational attainment influence public engagement with ehealth.^23^

These studies illustrate how the choice of measure of social position has important implications in assessing the equity impact of digital technologies. Furthermore, these are largely immutable factors and efforts to change the mediating role of these determinants are likely to be generational and possibly quasi-permanent in nature.

A second set of variables relates to socio-economic determinants, such as education, income and insurance or employment status. Digital skills measured by computer self-efficacy have been found to be an important determinant of online health information use in older adults.^22^^,^^24^

Finally, national economic development and geographic location are a third set of structural factors that determine usage patterns of digital technologies, which could also have a determining impact on health equity.^22^

A review of digital technology projects implemented in low- and middle-income countries found some evidence that digital health can positively influence health equity. One of the important channels for this to occur is the strengthening of upward and downward accountability. The authors of this review also highlight the need to include gender and power dimensions in any analysis of health equity.^25–27^ Furthermore, in developing countries with human resource poor settings, there is the potential for digital technologies to assist in the monitoring of patients who may otherwise be lost to follow up through gaps in the system.^28^ However, to date, the lack of necessary investment in ubiquitous information systems infrastructure means that inequitable digital advance has long been regarded responsible for the widening gap in the health and socioeconomic prosperity of many developing countries, particularly Africa’s resource poor nations.^29^

#### Personalized health technologies

There are a wide variety of systems, apps and technologies that all do very different things and their potential to reduce or exacerbate inequalities will differ depending on what is being referred to and whether the deployment is at the level of the individual or the system. Critical distinctions between technologies should be based on how, and in what context, these various technologies are both *accessed and used*.

Considerable effort has been made in recent years to create and promote apps and other digital technology-related tools in the area of health promotion, especially to aid lifestyle choices and behavior change. These are important advances and their impact on the capacity of health systems to respond and address conditions that affect human health cannot be overlooked or denied.^6^^,^^12^

Personalized health technologies that empower consumers to quantify health behaviors could advance health for all populations. The modifiable risk factors that are measured by these devices—including physical activity and diet—are major drivers of non-communicable diseases. These conditions, including cardiovascular and lung diseases, type 2 diabetes and various cancers, are the leading causes of death and disability worldwide.^2^^,^^5^

However, personalized health technologies are serving to widen inequities because only affluent early adopters can afford their higher prices, while marginalized populations remain excluded. Personalized health technologies further exacerbate inequities in the shorter term because early adopters are motivated and health conscious. Existing users tend to be highly educated and possess the necessary technological skills to operate the devices. They also have the linguistic and numeric capabilities to process information in order to change behaviors. Individuals of higher socioeconomic status (SES) are the first to adopt, and benefit most from, the introduction of innovative technologies in health, creating social inequalities in health where they were once very low or non-existent.^21–23^

### Applying health literacy as a lens to address the implications of digital technology on healthy equity

Of these factors, one of the greatest opportunities to affect change, in our view, lies in the opportunity to improve digital and health literacy simultaneously. Health literacy describes skills and competencies that enable people to gain access to, understand and apply health information to positively influence their own health and the health of those in their social environments. Research reveals that health literacy interventions which address health disparities and achieve, in one or more domains, reduction of disparities, also help increase health equity.^30^

In an increasingly media saturated and digitized world, these specific skill sets in the digital context are often referred to as e-health literacy or digital health literacy.^31^ This can be defined as the ability of people to use emerging information and communications technologies to improve or enable health and health care. These skills are increasingly necessary for accessing and navigating sources of health information and tools, such as television, the Internet and mobile apps.^32^

It has been shown that differences between persons classified as having high vs. low levels of e-Health literacy arise in terms of background attributes, information consumption and outcomes of the information search.^33^ The association of e-Health literacy with background attributes including traditional digital divide variables, such as sociodemographic characteristics, digital access and digital literacy indicates that the Internet reinforces existing social differences. Some people are being caught in a vicious cycle whereby a lack of digital access or the inability to make beneficial use reinforces and amplifies existing disadvantages often related to, for example, socio-economic factors.^34^

Individuals who are highly e-Health literate gain more positive outcomes from the information search in terms of self-management of health care needs and adoption of healthy behaviours.^35^ Highly literate users are also more likely to scrutinize information more carefully.^35^ This has implications in an era where fake news is known to travel faster and more widely than real news and evidence-based research.^36^ The more comprehensive and sophisticated use of the Internet and the subsequent increased gains among the high e-Health literate, therefore, create new inequalities in the domain of digital health information.

To bridge the gap the National Institute of Children’s Health Quality (US), for example, points out that the health literacy lens should be applied in everything they do to achieve the goals for health equity. As authors and producers of healthcare literature and digital information, it should always be made sure that information is not too complicated or too vague. Furthermore, a quality check is needed to see whether plain language is being used; materials provide clear instructions and next steps; and if processes and systems are set-up for reliable use or navigation by patients, families and various related professionals.^37^

A further example from Public Health England highlights that policymakers, public health agencies, schools, adult education services, employers, health and social care professionals and community groups, among others, all play a role in addressing health literacy, improving health outcomes and reducing inequalities.^38^ Promising health literacy strategies should be implemented to support people to take control of their, their families’ and their children’s health. In this regard, NHS England has been working with the Tinder Foundation, a social enterprise, to deliver digital skills training so that people with limited digital literacy skills, regardless of income, location, age, gender or ethnicity, can get online and access the support and information they need to improve their health, make informed choices and manage their own health more effectively. The project was developed in response to the fact that those most in need of NHS services are those least likely to have the skills they need to access online services.^38^ When combatting health inequality with health literacy strategies becomes a political priority it is possible to engage in wide-scale interventions that transform health systems.

The following framework can be used to develop digital technologies and assess their potential to affect equity. It proposes the evaluation of seven domains, namely: the ability to process information, engagement in own health, ability to engage actively with digital services, feeling safe and in control, motivation to engage with digital services, having access to information systems that work and digital services that suit individual needs. These empirically-derived domains form an e-health literacy framework and provide new insights into the user’s ability to understand, access and use e-health technologies.^39^

From an ethical standpoint, equity is emphasized, stressing the importance of accessible media environments for all—including those at risk of exclusion from (digital) media sources.^31^ The hidden algorithms, data-management systems and profiling operations that are a central part of the big data engine should not be allowed to foster processes that discriminate unjustly according to race, gender or SES. Furthermore, low-income groups should not be targeted to give up their data in exchange for access to products and services, as such ‘pay-for-privacy’ practices could create a new divide.^38^ Notably, the framework illustrates the need to work simultaneously on the personal, the contextual and the technological factors in order to mainstream the equity dimension. This will promote digital health as a tool to improve population health outcomes in an equitable fashion. There is a need to educate at-risk and needy groups (e.g. chronically ill) and to design technology in a mode befitting more consumers.

### Lessons learned, pitfalls and spring boards

As illustrated above, the issue of equity considerations around digital health appears to be gaining some traction and the healthy equity dimension is gaining ground both in research and in the discourse and policy debate on the future of digital health.

Applying lessons learned from the broader health technology diffusion, uptake and impact, we are likely to see a situation where over the short-term the inequalities associated with deployment of digital technologies will increase but as the uptake becomes more diffuse and experience increases, these inequalities would decline.^40^ The main difference is that whilst most traditional health technologies depended on a gatekeeper of health professional to gain access, with digital heath interventions there is a greater direct onus on patients. There is still a paucity of research and evaluation of digital technologies for health. Studies are small scale and context specific. The impact on equity and health inequalities is not often a consideration. The EU Expert Panel on Investing in Health called for the impact on equity to be taken into account in the framework developed for evaluation of digital health services.^12^ This remains a gap that needs to be addressed and traditional frameworks for assessing equity impact within health services may not fully capture the dimensions of digital technologies. It is for this reason that it is suggested that personal, social, literacy, contextual and technological factors are taken into account. The framework proposed above could be a step in starting to address this issue but it would need further work.

Policies may often end up exacerbating health inequalities unintentionally.^41^ For example, in the case of tobacco, the fact that quitters and non-smokers are more likely to be richer and better educated is intimated as a factor that has contributed to increasing health inequalities,^42^ yet nobody would advocate not pursuing activities that decrease tobacco smoking. One possible response could be to ensure that measures which as far as possible impact more greatly on the groups most in need alongside increasing health literacy are pursued simultaneously.

Firstly, further work on the development of methodologies and metrics to assess the impact of digital health on population health and health equity are needed. We trust that the perspectives in this article act as a starting point to clarify which aspect of health equity is being investigated. The move away from short term process indicators to health outcomes is necessary to inform the appropriate development of digital technology that is health and health equity enhancing.

Secondly, incorporating the use of digital health within population health approaches requires an understanding and acceptance that people come first and technology second. If digital health were to be construed as a ‘good’ using the concept of the commons,^43^ then it becomes possible to argue for the implementation of policies, regulatory environment, system architecture and industrial incentives be aligned in pursuit of that goal.^44^ Furthermore, alignment of micro and macro contextual spheres may contribute to facilitate both non-digital and digital media to effective support and promote public health.^45^

Thirdly, health equity can be promoted through attention to product development by employing the concept of frugal innovation, which heeds affordability and distinguishes between essential function and additional extras. Products should be invented based on health literacy design, co-creation and user-involvement. Using the universal precaution approach by default all will be benefit, not only people low on the health literacy spectrum.^46^

Looking to the future, it is heartening to see that the principles of solidarity and equity have made it into declarations such as the Montreal Declaration for the Responsible Development of Artificial Intelligence.^47^ However, governance frameworks at national and international levels have not kept up with the digital industry and in health systems in particular, there is still a belief that these can be fully governed at regional or national level with visible reluctance to relinquish regulatory authority to the level of the European Union.

## Conclusions

Health 4.0—which basically means the digital transformation of health and medical care, both in its practice and its governance––has its optimistic promoters and its pessimistic detractors. Irrespective of where public health professionals position themselves on this spectrum, it is of paramount importance to engage in order to safeguard the health of the most vulnerable.

It is pertinent to recall Julian Tudor Hart’s inverse care law,^48^ which states that health products and services are always used most by those who need them least. How do we turn this around? How do we ensure a robust research agenda to monitor impact and use the opportunity afforded by digital technology innovation to turn it into a force for reducing health inequity?

From an asset perspective, tapping into the digital world can help democratize people’s health through increased access to information regarding healthcare, disease prevention and health promotion. From a societal point of view, however, we owe it to everyone to build health literacy at all levels––individual, organizational, commercial, technical and political––so that people can withstand its inherent pitfalls.

*Conflicts of interest*: None declared.
